# Case Report: Primary Ovarian Leiomyoma: A Clinical Analysis of Case Series and Literature Review

**DOI:** 10.3389/fmed.2022.822339

**Published:** 2022-04-01

**Authors:** Guanmian Liang, Zaigui Wu, Li Zhu, Fei Ruan

**Affiliations:** ^1^Department of Nursing, Cancer Hospital of University of Chinese Academy of Sciences, Zhejiang Cancer Hospital, Hangzhou, China; ^2^Department of Gynecology, Women's Hospital, Zhejiang University School of Medicine, Hangzhou, China; ^3^Department of Ultrasound, Women's Hospital, Zhejiang University School of Medicine, Hangzhou, China

**Keywords:** leiomyoma, ovary, solid tumor, case series, surgical management

## Abstract

Ovarian leiomyoma accounts only for 0.5–1% of all benign ovarian tumors, and almost no preoperative diagnosis has been achieved. Commonly, these tumors are small in size and asymptomatic. However, they can become symptomatic as their size increases; thus, it is important to recognize this entity and differentiate them from ovarian malignant tumors. Radical surgeries with total hysterectomy and salpingo-oophorectomy are usually performed. However, ovary-preserving surgery has been suggested to young women, especially those desiring fertility in the future. In this study, we shared seven cases of primary ovarian leiomyomas and one inherent ligament leiomyoma, reviewed related articles to provide some new information about leiomyoma of the ovary, and discussed their proper surgical management.

## Introduction

Leiomyoma is one of the rarest ovarian benign tumors, accounting for 0.5–1% (1). Since the first case was identified in 1862 (2), fewer than 80 cases have been reported by case reports or case series. Most of them aged 17–79 years are asymptomatic and diagnosed incidentally during a routine pelvic examination, surgery, or even at autopsy (3). Symptoms include lower abdominal pain (4), ascites (5), pelvic mass, elevated cancer antigen 125 (CA125) level (6, 7), or Meigs' syndrome (8). Typically, ovarian leiomyoma is unilateral with no predilection for the right or left ovary, while bilateral cases have been reported mainly in women aged 16–25 and never in women aged more than 35 (4). Moreover, it is seldom diagnosed before operation due to low clinical incidence and difficulty in distinguishing it from a pelvic solid mass. Radical surgeries with adnexectomy or total hysterectomy and salpingo-oophorectomy are carried out, even in adolescent or young child-bearing women. In this study, we shared seven cases of primary ovarian leiomyoma and one inherent ligament leiomyoma to explore their proper surgical management. In addition, a review of related articles is presented.

This research was approved by the ethics committee of Women's Hospital Zhejiang University (No. 2019026), which confirmed that special approval was not required for this study. The authors agree to provide copies of the appropriate documentation upon request.

## Case Presentation

**Figure 1:**
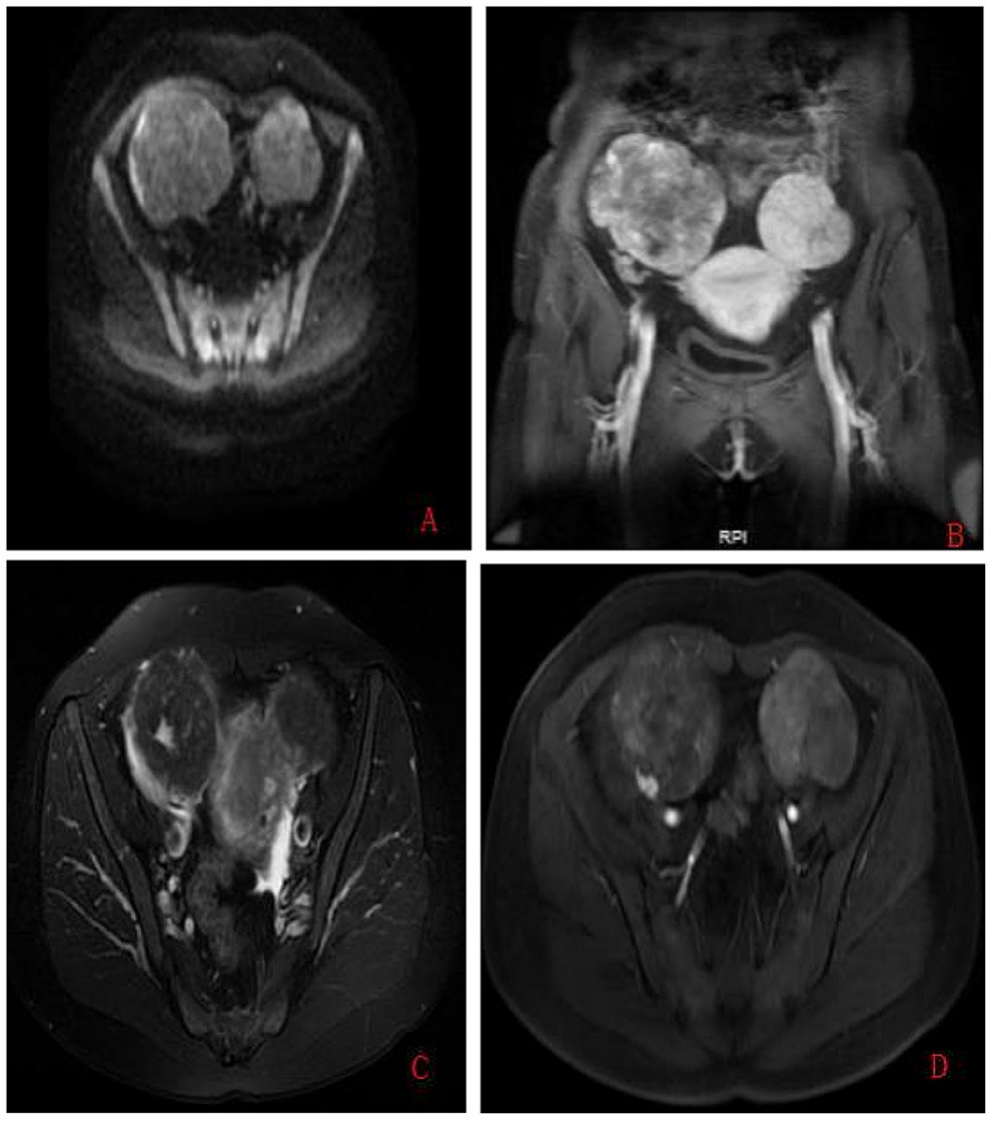
Preoperative pelvic MRI findings obtained for case 8. **(A)** On axial diffusion-weighted image (repetition time (TR)/echo time (TE): 4,000/72.4 ms, *b*-value = 500 s/mm^2^); the two masses showed the same intermediate signal intensity. **(B)** On sagittal T1-weighted with gadolinium administration (TR/TE: 3.9/1.7 ms), the left mass showed low signal intensity identical to the uterus myometrium. **(C)** On axial fat-saturated T2-weighted (TR/TE: 3,020/70.5 ms) image, the signal intensity of the left mass was continuous with the myometrium, while the right mass was well-circumscribed and sharply demarcated from the uterus. **(D)** On axial T1-weighted with gadolinium administration (TR/TE: 3.9/1.7 ms), flow voids were visible only between the tumor and the uterus myometrium on the left mass.

**Figure 2:**
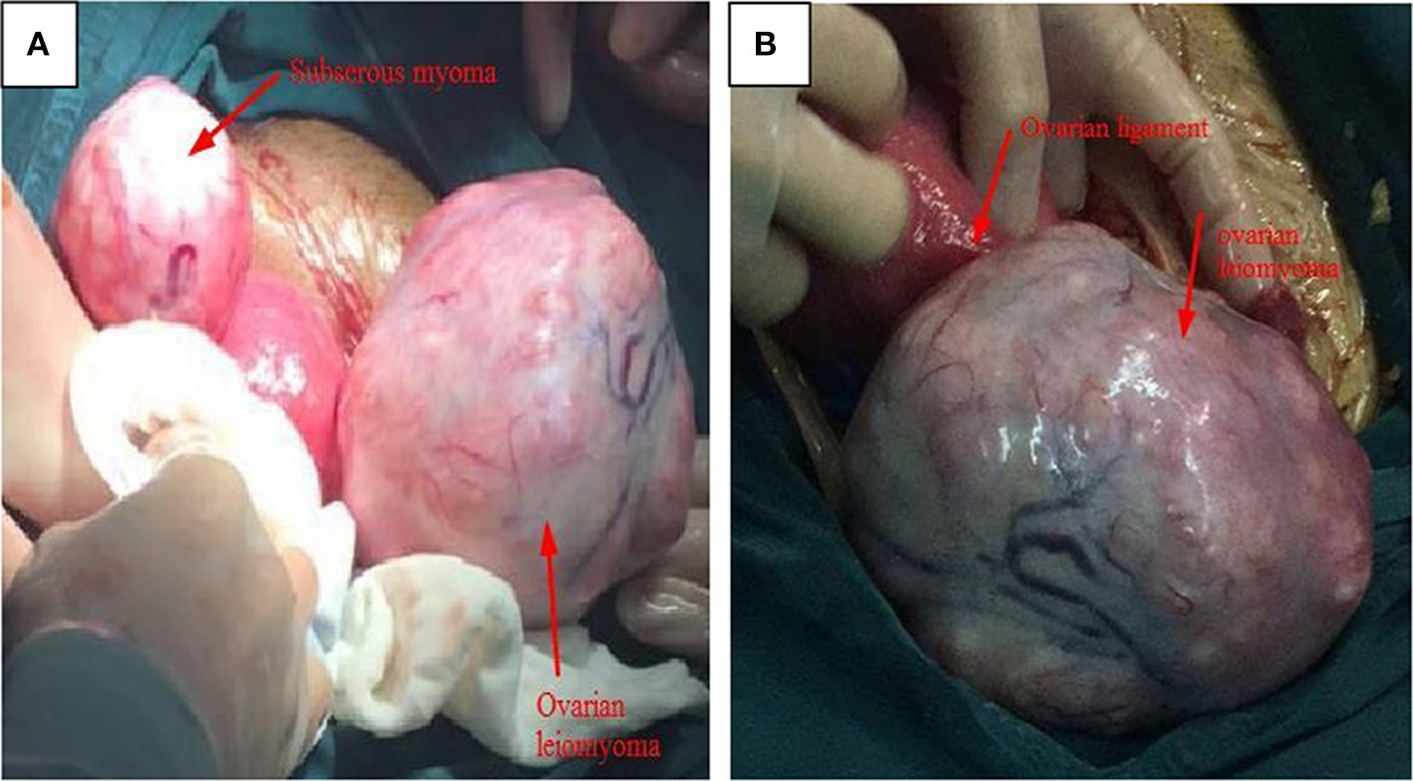
The gross characteristics of ovarian leiomyoma obtained for case 8. **(A)** Two solid, irregular, vascular-appearing mass was shown; the left growing from the uterine, while the other from the right ovary; the uterus was normal. **(B)** The thickened ovarian inherent ligament and enlarged right ovary with a sharply demarcated border, no adhesion, and no infiltration of the surrounding structures.

We reported a case series of seven primary ovarian leiomyomas and one inherent ligament leiomyoma diagnosed in the Pathology Department of Women's Hospital, Zhejiang University School of Medicine from 2012 to 2018. Clinical data were collected mainly by a search of medical records and are summarized in Tables 1, 2. The preoperative pelvic MRI findings and the gross characteristics of ovarian leiomyoma obtained for case 8 are also shown in Figures 1, 2.

**Table 1:** Demographic characteristics of our eight patients.

**Case**	**Age**	**Complaints/**	**Tumor**	**Tumor size**
	**(years)**	**Duration**	**location**	**(diameter, cm)**
				**on USG**
1	24	Pelvic mass/2 months	Right	6
2	65	Pelvic mass/20 years	Left	6
3	34	Pelvic mass/1 months	Left inherent ligament	3
4	57	Pelvic mass/1 months	Right	20
5	17	Vaginal bleeding/3 months	Left	8
6	29	Pelvic mass/1 months	Left	2
7	31	Primary infertility/4 years	Right	6
8	51	Pelvic mass/10 years	Right	8

**Table 2:** Surgical and histopathological results of our eight cases.

			**Histologic features**		
**Case**	**Preoperative diagnosis**	**Surgery method**	**Frozen section**	**Metotic index**	**Degeneration**
1	Sex cord-stromal tumor	Myomectomy	Leiomyoma	0	Hyaline, calcification
2	Subserous myomas	SOH	Leiomyoma	0	Hyaline, calcification
3	Ovarian tumor? myomas?	Myomectomy	Leiomyoma	0	None
4	Subserous myomas	SOH	Mesenchymal origin	0	Edema, hyaline
5	Sex cord-stromal tumors	Myomectomy	Mesenchymal origin	4/10HPF	None
6	Sex cord-stromal tumors	Myomectomy	Leiomyoma considered	0	None
7	Ovarian tumor? myomas?	Myomectomy	Fibroma or leiomyoma	0	None
8	Subserous myomas	Myomectomy	Leiomyoma	0	None

*SOH, salpingo-oophorectomy with hysterectomy; 10 HPF, number of mitotic figures per 400 high-power fields divided by 10*.

The mean age was 38.5 years (range 17–65 years). Six patients (75%) complained of an asymptomatic pelvic mass with the longest duration for 20 years, one had a history of primary infertility for 4 years and another presented with a 3-month history of irregular vaginal bleeding. The majority (62.5%) were located in the unilateral ovary, except one in the ovary inherent ligament. The mean diameter by ultrasound was 7 cm, and the CA125 level was normal in all cases.

**Figure 3:**
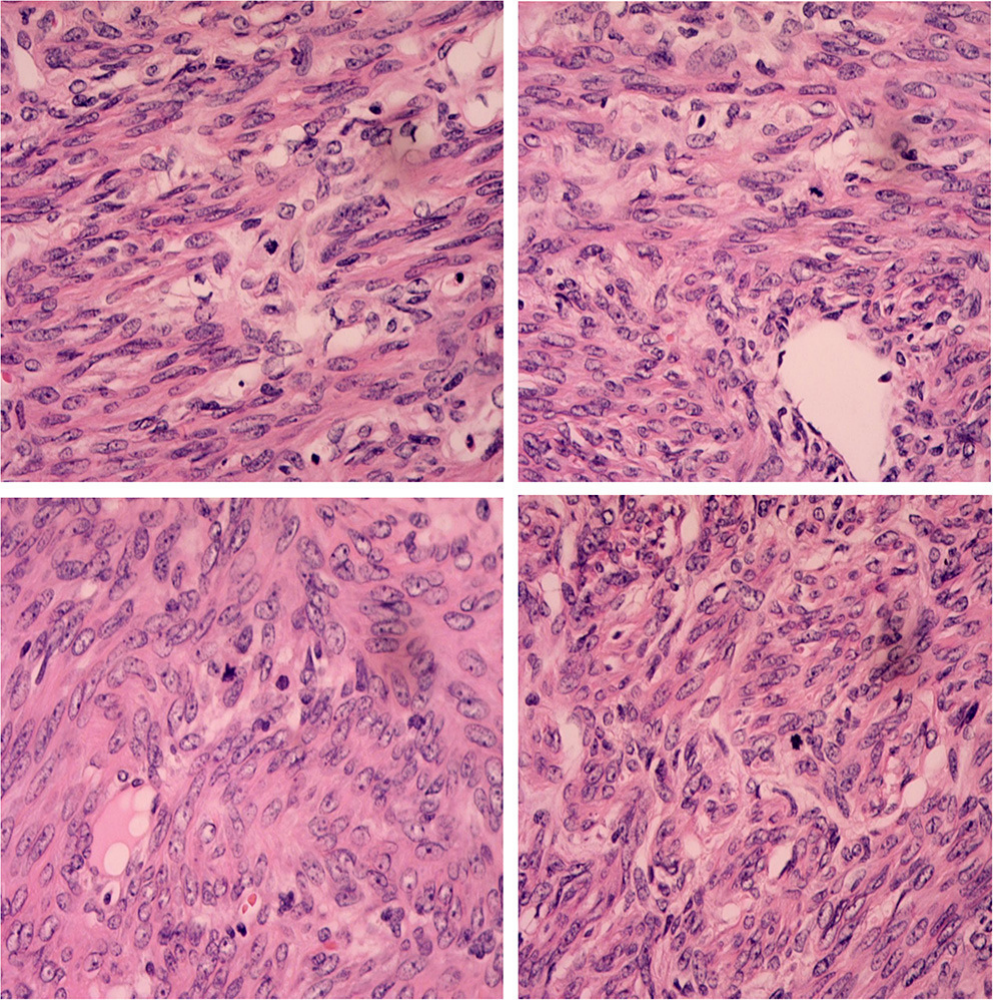
The histological images of case 5. Histological test results demonstrated ovarian leiomyoma with smooth muscle actin (SMA) positive and mitosis in 4/10 high-powered fields (H&E, original magnification 400 ×).

None of them was successfully diagnosed before the operation. Three (37.5%) were misdiagnosed as ovarian sex cord-stromal tumors and three (37.5%) as uterine subserous myoma, while the other two (25%) as ovary malignant tumors. The two oldest cases had a total hysterectomy and bilateral salpingo-oophorectomy, and a myomectomy was performed in the rest of the patients. Except for the 17-year-old girl who had 100 ml of ascites and nuclear division (4/10 high-powered fields [HPFs]), as shown in Figure 3, no other malignant cases, including cellular atypia, pleomorphism, and necrosis, were found. The surgical and histopathological results are shown in Table 2. All patients were followed up *via* telephone before the article was written, and none of them had tumor recurrence or surgery complications.

## Results of Literature Review

**Figure 4:**
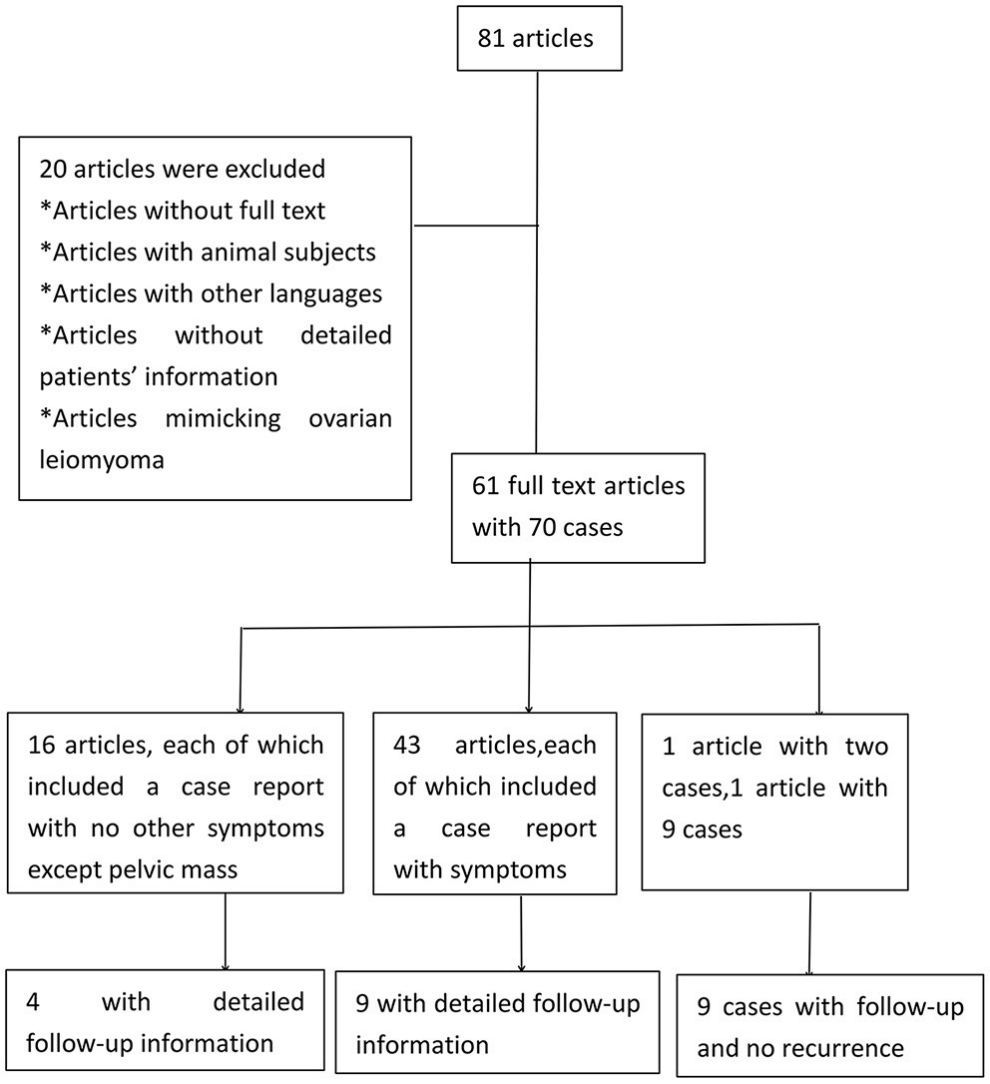
Chart of the literature electronic search of ovarian leiomyoma.

A PubMed search for case reports and series was also conducted with “ovarian leiomyoma” or “smooth-muscle tumors of the ovary” used as keywords. The literature inclusion criteria were human subjects, written in English, and full-text availability, while editorial and conference highlights were excluded. The patients' data were extracted, and the clinical data were described or summarized. Only cases with detailed demographic characteristics and surgical procedures were analyzed, excluding tumors without proven pathological diagnoses. The whole screening process was independently completed by Guanmian Lang and Li Zhu, while Zaigui Wu and Fei Ruan was consulted for any disagreements. A total of 61 full-text articles with 70 cases were included for analysis (Figure 4).

## Clinical Features of Ovarian Leiomyoma

As summarized in Tables 3, 21 cases were asymptomatic, except pelvic mass, and their mean age was 41.43 years with a range of 17–84 years. They were incidentally found using ultrasound or during abdominal surgeries. A total of 49 patients presented mainly with abdominal pain, of whom the youngest was 13 years and the oldest was 103 years with a mean age of 45.56 years. Apart from the main complaint, other rare presentations including vaginal discharge, virilization, and menstrual abnormality have also been reported. None of the 70 cases were diagnosed correctly before surgery, and the most common misdiagnosis was uterine myoma. The second most common misdiagnosis was malignant tumors and fibroma/thecoma, with only a small number of cystadenoma and teratoma misdiagnosed.

**Table 3:** Age distribution and preoperative diagnosis of ovarian leiomyoma.

**Cases**	**Age (** ***y*** **)**			**Initial diagnosis**						
	** <35**	**35–45**	**≧45**	**UL**	**Fibroma/**	**Cystadenoma/**	**OM**	**ST**	**MT**	**UD**
					**thecoma**	**teratoma**				
Asym^a^	8	5	8	7	4	1	6	0	0	3
Sym^b^	15	6	27	11	4	4	13	4	10	3

*U, uterine leiomyoma; OM, ovarian mass; ST, solid tumor; MT, malignant tumor; UD, cases without diagnosis. ^a^ is the number of asymptomatic patients and ^b^ is the number of symptomatic individuals*.

## Laboratory Findings of Ovarian Leiomyoma

Ovarian leiomyoma with increased CA125 levels was rare, and information about each case report is summarized in Table 4. It was more common in postmenopausal women with a predilection for the left ovary. An elevated CA125 level with the adnexal mass was suggestive of ovarian malignancy, and thus total hysterectomy with salpingo-oophorectomy was usually performed. One of the reported cases had a level even up to 4, 877 U/ml, and a more invasive surgery was carried out.

**Table 4:** Characteristics of ovarian leiomyoma with an elevated CA125 level.

**Author**	**Age (*y*)**	**Clinical presentations**	**CA125 (U/ml)**	**Preoperative diagnoses**	**Surgery method**	**Site**	**Size (cm)**	**Histologic features**	**Post-operative course**
Ichigo et al. (9)	76	Abdominal discomfort	49.9	Broad ligament leiomyoma	TAH + BSO	R	19*11*10	Mitotic 2-3/10HPF, Cystic, hyaline, myxomatous	Uneventful
Sasikala et al. (7)	53	Abdominal pain, distension	50	Ovarian fibroma	TAH + BSO + omental biopsy	L	16*12	Sclerosis	Uneventful
Kurai et al. (8)	76	Chest pain, dyspnea, pleural effusion	163	Ovarian tumor	TAH + BSO	L	9*7*7	Mitotic 1–2/10 HPF, Meigs'syndrome	Pleural effusion disappeared, CA-125 normal
Erdemoglu et al. (10)	45		70.6	Fibroma /thecoma	TAH + BSO	L	8*9	None	
Yumru et al. (6)	55	Epileptic seizure, abdominal pain	4, 877	Ovarian tumor	TAH + BSO + lymphadenectomy + appendectomy+ infracolic omenectomy + multiple biopsies	L	30*28*15	Unknown	No more epileptic seizures

*TAH, total abdominal hysterectomy; BSO, bilateral salpingo-ophorectomy; R, the right ovary; L, the left ovary*.

Ovarian leiomyoma associated with abnormal hormonal production was extremely rare, and the data are summarized in Table 5. Three of the cases were detected due to androgen overproduction such as male-type baldness or excessive hair growth. These leiomyomas can induce theca cell's luteinization. The elevated testosterone decreased dramatically, and manifestations of overt androgen improved markedly after tumorectomy. Amenorrhoea and abnormal uterine bleeding were also observed in reproductive or postmenopausal women with ovary leiomyoma, which might be due to inhibin B and luteinizing hormone produced by the tumor.

**Table 5:** Characteristics of ovarian leiomyoma with abnormal hormonal synthesis.

**Author**	**Age (*y*)**	**Clinical symptoms**	**Abnormal Tests**	**Preoperative diagnoses**	**Surgery method**	**Tumor site**	**Size (cm)**	**Histologic features**	**Post-operative course**
Parish et al. (11)	68	Virilization	T 1082 ng/dl, free T 25.8 ng/dl	Ovarian mass	TAH + BSO	Right	10*10*4.5	Hilus cells proliferation	T 45ng/dl on first postoperative day. died 1 month letter for cerebral infarction.
Mallya et al. (12)	56	Excessive hair growth	T 49 nmol/l	Ovarian mass	TAH + BO	Left	11*11*7	Theca cell reaction, edema, fatty tissue	T normal; markedly improved scalp hair growth; facial and truncal hair reduced considerably; clitoromegaly regressed postoperation 3 months
Carpén et al. (13)	64	Hair loss, male type baldness	T 20.4 nmol/l	Ovarian mass	BO	Right	7*5*5	Luteinized theca cells	T decreased dramatically; hair growth not weaken
Abdel-Gadir et al. (14)	35	Amenorrhoea, infertility	Inhibin B 408 pg/ml, LH 19.5 IU/l	Broad ligament leiomyoma/ ovarian fibroma	Mass excision	Right	7.4*4.1*6.1	Parasitic leiomyoma	menstruation recovered, uterine enlarged, natural conception;successful pregnancy by IVF

*T, testosterone; BO, bilateral oophorectomy; IVF, in vitro fertilization*.

## Surgery Method of Ovarian Leiomyoma

Surgical procedures for cases of ovarian leiomyoma are shown in Table 6. In cases where patients aged less than 35, 10 had unilateral oophorectomy or salpingo-oophorectomy and 11 had mass excision, while one aged 17 had bilateral salpingo-oophorectomy (3) and another aged 29 had total hysterectomy and bilateral salpingo-oophorectomy (15). In women aged between 35 and 45 years, unilateral oophorectomy/salpingo-oophorectomy was performed in five patients, bilateral salpingo-oophorectomy in one patient, and total hysterectomy and unilateral salpingo-oophorectomy in two patients, while mass excision was performed in three patients. In individuals more than 45 years, 21 had total hysterectomy and bilateral/unilateral salpingo-oophorectomy, 5 had bilateral oophorectomy/salpingo-oophorectomy, 9 had unilateral oophorectomy/salpingo-oophorectomy, whereas only one had mass excision.

**Table 6:** Surgical procedures for cases of ovarian leiomyoma.

**Surgery**	**Age** **<** **35y**		**35y** **>** **Age** **<** **45y**		**Age** **≧45y**	
	**Asymptomatic**	**Symptomatic**	**Asymptomatic**	**Symptomatic**	**Asymptomatic**	**Symptomatic**
UO/USO	3	7	2	3	2	6+^1^
Mass excision	4	7	1	2	0	1
TH+BSO/USO	0	1	2	0	4	17
BO/BSO	1	0	0	1	2	3

*UO, unilateral oophorectomy; USO, unilateral salpingo-oophorectomy; TH, total hysterectomy*.

*+1Left SO and right myomectomy were performed in a 25-year-old case with bilateral ovarian leiomyoma*.

## Tumor Site, Size, and Histological Features of Ovarian Leiomyoma

In Table 7, a predilection for the right ovary, especially in symptomatic cases, is noted. In a total of 63 unilateral cases, 27 leiomyomas were located in the left ovary and 35 in the right ovary. Of the six bilateral cases, six were aged below 35 years, one aged 45 years, and the other aged 46 years. Mass with a diameter of more than 10 cm was observed in nearly half, both in asymptomatic and symptomatic individuals. Seven asymptomatic and 18 symptomatic patients had a tumor diameter between 5 and 10 cm. Hyalinization and edema were the most common types of degeneration, while myxoid degeneration was much more common in symptomatic cases. Symptomatic cases also had mitotically active, atypical cells.

**Table 7:** Tumor site, size, and histopathological results of patients diagnosed with ovarian leiomyomas.

**Case**	**Site**			**Size (cm)**			**Risk**			**Degeneration**					
	**L**	**R**	**Bi**	**≧10**	**5–10**	** <5**	**MI**	**Atypia**	**Necrosis**	**Hyaline**	**Cystic**	**Myxoid**	**Edema**	**Hem**	**Cal**
Asy^a^	9	10	2	10	7	4	0	0	1	7	2	1	3	1	2
Sym^b^	20	25	4	22	18	7	7	3	1	9	5	7	4	5	0

*Bi, bilateral ovary; MI, mitotic index; Hem, hemorrhage; Cal, calcification; a, Asymptomatic cases; b, Symptomatic cases*.

## Follow-Up After Operation

Follow-up information was available for 22 cases in 13 case reports (Table 8) and one case series (29). During the follow-up period (range 1–48 months), only one case had tumor recurrence (19). She was a 24-year-old nulliparous woman who first had left oophorectomy and omentectomy with right ovary biopsies for a left ovarian cyst sized 9 cm ×8 cm × 11 cm and some small nodules (<1 cm) on the right ovary. Nuclear pleomorphism with high mitotic activity in one area was indicated, but this was insufficient to diagnose a sarcoma by an external histological center. Increasing abdominal pain and girth occurred at 7 months and a multiple cystic ovarian mass measured 5 cm × 3 cm was found in the right ovary. The second pathological examination showed extensive cystic destruction with some solid leiomyoma areas, but no ovarian tissue remaining.

**Table 8:** Characteristics of ovarian leiomyoma with detailed follow-up information.

**Author**	**Age(y)**	**Complaints**	**Preoperative diagnoses**	**Surgery Method**	**Site**	**size(cm)**	**Histologic Features**	**Follow-up**
Sapala et al. (16)	103	Abdominal pain	Malignant tumor	Mass excision	L	30*30*19.5	cellularity	2 years, no recurrence
Zhao et al. (17)	28	Abdominal pain	Adnexal tumor	Mass excision	R	10.4*10*6.6	Ischemic infarction	3 years, no recurrence
Shrestha et al. (18)	25	Abdominal pain	Malignant tumor	Left USO + right myomectomy	Bi	18*5(left), 5*3(right)	Edema	9 months, no recurrence
Emovon et al. (19)	24	Abdominal pain, nausea, vomiting	Malignant tumor	LO + omentectomy	L	9*8*11	Pleomorphism with focal high mitotic activity	7 months, recurrence
Alshwairikh et al. (20)	45	Abdominal pain, distension	Peritoneal malignancy	LO + omentectomy	L	39*30.2	Cystic degeneration	6 months, no recurrence
Kitamura et al. (21)	50	Excessive menstruation	Ovarian tumor	TLH + USO	R	5.5*3*3	Wide fibrosis, hyalinization	6 months, no recurrence
Kim et al. (22)	35	Acute abdomen, pregnancy 10gw	Torsion of pedunculated myoma	USO	R	9.3*7.8, twisted	None	Delivered at 40 + 1 gw, no recurrence
Tomas et al. (23)	31	Abdominal pain, fever, vomiting	Acute appendicitis	USO + appendectomy	L	11*10*6	Endometriotic cyst, periappendicitis	6 months, no recurrence
Wang et al. (24)	58	Abdominal distention	Ovarian solid tumor	USO	R	20*18	Atypical leiomyoma	9 months, no recurrence
Daniel et al. (25)	31	Term CS	Unknown	Mass excision	Bi	multiple small tumors	Calcification	2 months, no recurrence
Sato et al. (26)	61	Mass screening	Ovarian tumor	TAH + BSO	R	16.0*10.0*5.0	Cystic degeneration	2 years, no recurrence
Kohno et al. (27)	30	Threatened abortion at 16gw	Uterine leiomyoma	Mass excision	L	23*23*20	Hyalinization, edema	Delivery at 40gw, no recurrence
Raje et al. (28)	31	Incidental finding	Ovarian fibroid	Mass excision	R	5*4*4	None	1 year, no recurrence

*CS, cesarean section; LO, left oophorectomy; TLH, total laparoscopic hysterectomy*.

## Discussion

In this article, we reported eight cases of leiomyoma from the ovary or ovarian inherent ligament. In the literature review, we found that leiomyoma of the ovary has a predilection for the right ovary, can attain massive sizes and cause pressure symptoms, and can be bilateral in women over 35 years, which seems to be in contradiction with previous reports. In addition, ovarian leiomyoma demonstrates an extremely low risk of malignant degeneration, and efforts should be made to preserve fertility and improve the life quality by avoiding destructive ovarian surgery.

Ovarian leiomyoma can occur in women of all ages, but nearly 80% is found in patients aged 20–65 years (30). So far, only five cases (7.1%) aged <20 years have been reported, and in our study, only one (12.5%) is below this age. It has been revealed to rarely exceed 3 cm in diameter (31). However, most reported cases have a diameter of more than 5 cm, and some even have a mass extending higher than umbilicus, as summarized in this review. An obvious predilection for the right ovary has been observed too. Thus, ovarian leiomyoma may not be small in size and is often unilateral but with a right ovary predilection.

More than two-thirds of the reviewed cases are symptomatic. Clinical manifestations include pelvic pain, abnormal uterine bleeding, menorrhagia, and frequent urination, which are similar to uterine fibroid and associated with tumor size (32, 33). Their relationship with infertility has been reported (29), and one of our cases indeed had 4 years of infertility. Virilization arises from elevated testosterone levels second to theca cell proliferation (11–13), while appendicitis-like symptoms might result from periappendicitis (23, 34). Meigs' syndrome is very rare, and only two cases have been reported in 2005 (8) and 2006 (10). The etiology of these rare presentations is not known and might be associated with tumor origin.

Symptoms of ovarian leiomyoma are diverse and nonspecific, whereas the presence of imaging features and laboratory findings are nonspecific as well. Preoperative evaluations by ultrasound, CT, or MRI have difficulty in demonstrating its origin or distinguishing them from other pelvic solid tumors. Their cystic, hemorrhagic, and calcified degeneration on radiography may even suggest malignant risk, especially in combination with high CA125 levels or ascites, resulting in a more invasive surgery (6). Frozen section examination is helpful in determining the extent of surgery, and its accuracy for leiomyoma of the ovary in our investigation ranges from 50 to 62.5%.

Ovarian leiomyoma can be diagnosed by a combination of histopathological evaluation and immunohistochemical markers; however, caution should be taken when distinguishing with fibroma using smooth muscle actin (SMA) (35). Significant nuclear atypia, pleomorphism, and necrosis have been traditionally utilized to evaluate ovarian malignant leiomyoma; however, mitotic activity, necrosis, and atypical cells need to be evaluated carefully as these tumors can also have a benign course (36). A high mitotic activity in the presence of nuclear pleomorphism is an indication of potential malignancy, and these patients tend to be recurrent.

As summarized in this review, total hysterectomy and bilateral salpingo-oophorectomy are performed usually in middle-aged and older patients, and this surgical procedure has been performed in only one patient aged 29 (15). Unilateral salpingo-oophorectomy or oophorectomy is a better choice for all ages of patients with unilateral leiomyoma, while bilateral oophorectomy has often been required for those with bilateral leiomyoma as reported earlier (3). However, mass excision or ovarian sparing procedures have been gradually reported especially in younger cases, and no recurrence has been reported so far. Thus, an ovary-preserving surgery has been suggested for women who desire fertility, unless potential malignancy is observed in the tumor. Ovarian leiomyoma has a pseudocapsule similar to those from the uterus, and intracapsular myomectomies can be performed easily. To date, no studies have assessed the margin status and the only one recurrent patient first had right ovarian biopsies for some small nodules <1 cm, whereas the second histological examination showed extensive cystic destruction and some solid leiomyoma areas with no ovarian tissue remaining.

In conclusion, primary ovarian leiomyoma is mostly benign in nature with an excellent prognosis. The follow-up of patients with a smaller tumor size is an option, but radical surgical treatment has been suggested for those with a mass diameter exceeding 3–4 cm to avoid ovarian destruction by the tumors. Mass excision is preferred in women of reproductive desire, whereas total hysterectomy and bilateral salpingo-oophorectomy can be considered in middle-aged and older adult patients.

## Author Contributions

ZW collected the clinical data, drafted, and revised the manuscript. GL and LZ screened and summarized the review literature. GL helped to draft the manuscript and revised the manuscript. FR drafted and revised the manuscript. All authors read and approved the final manuscript.

## Funding

This research was supported by the Natural Science Foundation of Zhejiang Province (No. LQ19H040011 and LGF22H040002), the Zhejiang Province Medical and Health Technology Project (No. 2019KY431), and the Program for Higher Education Research Association of Zhejiang University (No. G2127).

## Conflict of Interest

The authors declare that the research was conducted in the absence of any commercial or financial relationships that could be construed as a potential conflict of interest.

## Publisher's Note

All claims expressed in this article are solely those of the authors and do not necessarily represent those of their affiliated organizations, or those of the publisher, the editors and the reviewers. Any product that may be evaluated in this article, or claim that may be made by its manufacturer, is not guaranteed or endorsed by the publisher.
